# Varicella zoster virus productively infects human natural killer cells and manipulates phenotype

**DOI:** 10.1371/journal.ppat.1006999

**Published:** 2018-04-30

**Authors:** Tessa Mollie Campbell, Brian Patrick McSharry, Megan Steain, Thomas Myles Ashhurst, Barry Slobedman, Allison Abendroth

**Affiliations:** 1 Discipline of Infectious Diseases and Immunology, The University of Sydney, Sydney, New South Wales, Australia; 2 Sydney Cytometry Facility, The University of Sydney, Sydney, New South Wales, Australia; 3 Discipline of Pathology, The University of Sydney, Sydney, New South Wales, Australia; Emory Vaccine Center, UNITED STATES

## Abstract

Varicella zoster virus (VZV) is a ubiquitous human alphaherpesvirus, responsible for varicella upon primary infection and herpes zoster following reactivation from latency. To establish lifelong infection, VZV employs strategies to evade and manipulate the immune system to its advantage in disseminating virus. As innate lymphocytes, natural killer (NK) cells are part of the early immune response to infection, and have been implicated in controlling VZV infection in patients. Understanding of how VZV directly interacts with NK cells, however, has not been investigated in detail. In this study, we provide the first evidence that VZV is capable of infecting human NK cells from peripheral blood *in vitro*. VZV infection of NK cells is productive, supporting the full kinetic cascade of viral gene expression and producing new infectious virus which was transmitted to epithelial cells in culture. We determined by flow cytometry that NK cell infection with VZV was not only preferential for the mature CD56^dim^ NK cell subset, but also drove acquisition of the terminally-differentiated maturity marker CD57. Interpretation of high dimensional flow cytometry data with tSNE analysis revealed that culture of NK cells with VZV also induced a potent loss of expression of the low-affinity IgG Fc receptor CD16 on the cell surface. Notably, VZV infection of NK cells upregulated surface expression of chemokine receptors associated with trafficking to the skin –a crucial site in VZV disease where highly infectious lesions develop. We demonstrate that VZV actively manipulates the NK cell phenotype through productive infection, and propose a potential role for NK cells in VZV pathogenesis.

## Author summary

Varicella zoster virus (VZV) is a pervasive pathogen, causing chickenpox during primary infection and shingles when the virus reactivates from latency. VZV is therefore a lifelong infection for humans, warranting investigation of how this virus interacts with the immune system. One of the first immune cells to respond to viral infection are natural killer (NK) cells, yet little is known about how VZV interacts with NK cells. We demonstrate for the first time that VZV infects human blood NK cells and can use them to pass on infection to other cells in culture. Furthermore, VZV displays a predilection for infecting mature NK cells, and amplifies expression of receptors that would promote trafficking to the skin– the site of highly infectious lesions during chickenpox and shingles. Our findings suggest a role for NK cells in VZV disease and enhances our understanding of how lifelong infections interact with the human immune system.

## Introduction

Varicella zoster virus (VZV) is a human alphaherpesvirus with worldwide prevalence. VZV is responsible for varicella during primary infection and herpes zoster following reactivation from latency in sensory ganglia. Infection caused by VZV places a substantial burden on healthcare systems throughout the world, despite the availability of vaccination [1, 2]. Primary infection with VZV is regarded as beginning in the upper respiratory tract where infection of epithelial cells allows the virus to breach lymphoid tissue and infect immune cells such as T cells and diverse subsets of dendritic cell (DCs) [3–5]. Subsequent viremia and dissemination of virus to internal organs and the skin during the prolonged incubation period (typically 14–16 days) is considered to be facilitated by the trafficking of infected T cells [6]. This model of pathogenesis is supported by clinical studies of non-immunocompromised patients with varicella, where VZV could be cultured from peripheral blood mononuclear cells (PBMCs) isolated during the incubation phase of disease and peaking before the onset of vesicular rash [7, 8]. Subsequent reports were able to identify infected cells with lymphocyte morphology [9, 10] and later studies sought to confirm infection of T cells and B cells [11–13], overlooking the third major lymphocyte population in peripheral blood– natural killer (NK) cells. The delayed development of the NK cell field [14] in comparison to our understanding of T cell and B cell immunology most likely accounts for these earlier reports overlooking a possible role for NK cells.

It is surprising that there has been little investigation into discerning the interaction of VZV with NK cells given clear evidence that they are involved in VZV pathogenesis. Clinical studies of patients with NK cell deficiencies have reported disseminated varicella with multi-organ complications [15, 16], that was ultimately fatal in some cases [17, 18]. Additionally, the potential for NK cells to play a part in viral dissemination during primary infection is evident from a case of severe, persistent varicella, where VZV DNA was detected in peripheral blood NK cells, as well as T cells and B cells [19]. These reports make a firm argument for the involvement of NK cells during VZV infection. As innate immune lymphocytes that are early responders to infection, NK cells circulate through the blood, localise to lymphoid tissue, and have the ability to migrate to inflamed tissue at distal sites of the body [20]. NK cells are thus well positioned to be involved in controlling VZV infection, as well as possibly disseminating virus. In contrast to the exquisite specificity of T cell and B cell responses to a specific antigen, NK cells use a multitude of germline-encoded receptors to assess the state of a cell. Detection of infection or deleterious transformation leads to cytolytic killing of the cell, as well as secretion of proinflammatory cytokines [21]. We have previously demonstrated, however, that VZV significantly modulates expression of cell-surface ligands to the NK cell activating receptor, NKG2D, and that NK cells do not display enhanced degranulation against VZV infected cells [22].

NK cells are extremely heterogeneous, with immense phenotypic diversity between individuals as well as within each individual [23]. Overarching this diversity, NK cells can be divided into two major subsets: the CD56^dim^ population which dominate the pool of NK cells circulating in the blood, and CD56^bright^ NK cells which are more abundant in lymph nodes and tonsils [24]. More recent findings suggest that CD56^dim^ NK cells develop from the CD56^bright^ population [25–27]. These NK cell subsets also differ in their expression of the low-affinity IgG Fc receptor CD16, which is predominantly expressed on CD56^dim^ NK cells and can mediate cytolytic activity against antibody-coated target cells through antibody-dependent cell-mediated cytoxicity (ADCC) [28]. It is believed that the NK cell pool of an individual will adapt and change over a lifetime [29], especially in the frequency of CD56^dim^ NK cells expressing the terminally sulphated glycan carbohydrate CD57. This marker is considered to be an indicator of mature NK cells given that frequency of expression increases with age [30, 31], and the observations that expression is absent on foetal and newborn NK cells [32], as well as CD56^bright^ NK cells [33, 34]. Mature CD57^+^CD56^dim^ NK cells have been characterised as having decreased expression of NKG2A, high levels of CD16, and increased acquisition of inhibitory LIR-1 and killer cell immunoglobulin-like receptors (KIRs) [35, 36]; the cumulative changes fine-tuning NK cells to their environment.

Given the interactions between NK cells and viruses during the course of the immune response to infection, it is not surprising that several human viruses have developed the ability to infect NK cells. Amongst the herpesvirus family to which VZV belongs, NK cell infection has been demonstrated *in vitro* with herpes simplex virus [37, 38], Epstein-Barr virus [39], and human herpesvirus 6 [40]. Additionally, human immunodeficiency virus has been shown to productively infect CD4^–^ and CD4^+^ NK cells [41, 42], whilst influenza virus and vaccinia virus have both been found to establish non-productive infections in NK cells [43, 44]. From these studies, it is apparent that predominantly chronic viral infections have evolved NK cell tropism, however, to the best of our knowledge it has not been investigated whether NK cells are permissive to infection with VZV.

Here, we demonstrate for the first time that human peripheral blood NK cells support productive VZV infection and are capable of transmitting virus *in vitro*. VZV preferentially infected the mature CD56^dim^ NK cell population, and analysis of maturity markers revealed acquisition of CD57 expression driven by VZV infection, while culture with VZV led to a loss of cell-surface CD16. Additionally, we show that VZV infected NK cells upregulate skin-homing chemokine receptors, inducing a phenotype that could facilitate dissemination of virus in the host. The findings presented here provide insight into viral influence on NK cell phenotype, and hold significant implications for our understanding of VZV pathogenesis, proposing NK cells as potential key players in mediating VZV disease.

## Results

### Varicella zoster virus is able to infect human NK cells, CD3^+^CD56^+^ lymphocytes and T cells in peripheral blood

**Fig 1:**
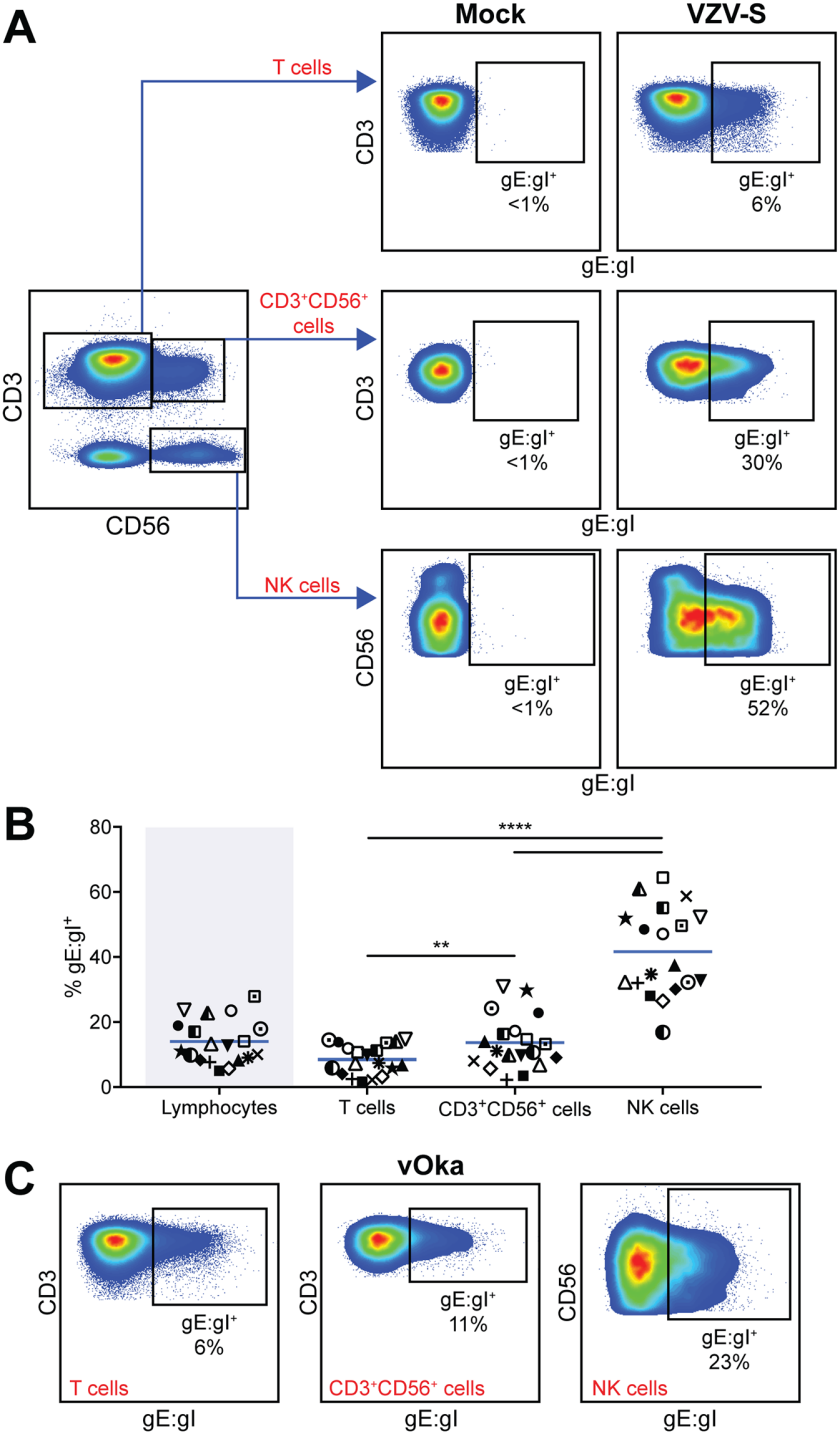
VZV infects human peripheral blood NK cells, CD3^+^CD56^+^ lymphocytes, and T cells. Healthy human donor PBMCs were inoculated with mock or VZV infected ARPE-19 epithelial cells for 2 days then analysed for infection by flow cytometry. (A) Representative flow cytometry plots of mock or VZV-S infection, examining surface VZV gE:gI expression on live T cells (CD3^+^CD56^–^), CD3^+^CD56^+^ lymphocytes, and NK cells (CD3^–^CD56^+^). (B) Frequencies of live gE:gI^+^ lymphocytes in total (shaded), compared to specific populations: T cells, CD3^+^CD56^+^ lymphocytes, and NK cells (n = 19). Symbols represent individual donors consistent across lymphocyte populations, and bars indicate mean. Statistical analysis was performed between specific lymphocyte populations. **p < 0.01, ****p < 0.0001 (RM one-way ANOVA with the Greenhouse-Geisser correction and Tukey’s multiple comparisons test). (C) Representative flow cytometry plots of vOka infection, examining surface gE:gI expression on live T cells (CD3^+^CD56^–^), CD3^+^CD56^+^ lymphocytes, and NK cells (CD3^–^CD56^+^) (n = 3).

The capacity of VZV to infect human T cells is well established [45] however to the best of our knowledge, the permissiveness of NK cells or CD3^+^CD56^+^ lymphocytes to VZV infection has not been characterised. To initiate investigation, we looked for transmission of infection to human PBMCs through coculture with ARPE-19 epithelial cells that had been infected with a clinical isolate of VZV (VZV-S). A cell-associated model of infection was used to transfer infection to PBMCs as VZV is highly cell-associated *in vitro* [46], and this method is an established technique for infection of human immune cells with VZV [3, 4]. By examining expression of the VZV surface glycoprotein complex glycoprotein E: glycoprotein I (gE:gI) on live lymphocytes by flow cytometry, we confirmed an average infection of 8% of T cells (range: 2–15%) (Fig 1A and 1B), consistent with previous reports [4]. We also found gE:gI expression on an average of 14% of CD3^+^CD56^+^ lymphocytes (range: 2–31%) and 42% of NK cells (range: 17–65%) (Fig 1A and 1B), indicating VZV infection of these cell types for the first time. In comparison to these specific cell populations, infection was transmitted to an average of 14% of the total lymphocyte pool (range: 5–30%) (Fig 1B). Across the 19 donors examined, NK cells consistently showed significantly higher levels of infection, more than 5-fold greater than T cells and 3-fold above CD3^+^CD56^+^ lymphocytes (Fig 1B).

In addition to determining infection by surface staining for gE:gI, we compared cell-associated infection of human PBMCs using a GFP-expressing strain of VZV (VZV-GFP). Detection of GFP^+^ T cells, CD3^+^CD56^+^ lymphocytes and NK cells was comparable to levels of gE:gI staining (S1 Fig), lending further support to our observations of infection.

We next asked whether the efficiency of infection for these lymphocyte populations would be the same with the attenuated vaccine strain of VZV (vOka) given that it has previously been found to maintain the ability to infect T cells [47]. In comparison to virulent VZV-S, similar trends of gE:gI expression were found on T cells, CD3^+^CD56^+^ lymphocytes and NK cells using a cell-associated method of infection (Fig 1C). Using cell-free preparations of the attenuated vaccine (VARIVAX) we also observed infection of NK cells through gE:gI detection (S2 Fig). Measurement of infection 2 days after cell-free inoculation would include both cells initially infected by the cell-free VARIVAX, as well as subsequent spread of virus through the culture. Although efficiency of infection with VARIVAX was additionally variable, this finding indicates that NK cells are permissive to VZV infection through cell-free mechanisms.

Taken together, these results indicate that along with peripheral blood T cells, NK cells and a proportion of CD3^+^CD56^+^ lymphocytes can also be infected by both virulent and vaccine strain VZV, identifying novel lymphocyte populations permissive to infection by VZV.

### Stimulation of lymphocytes with IL-2 enhances VZV infection

Interleukin-2 (IL-2) is an immunomodulatory cytokine that is critical during infections for activating and directing lymphocyte function. Specifically in the case of varicella, IL-2 has been detected in the serum of varicella patients [48] and it has been demonstrated that VZV-specific T cells produce IL-2 upon stimulation with VZV lysate [49].

**Fig 2:**
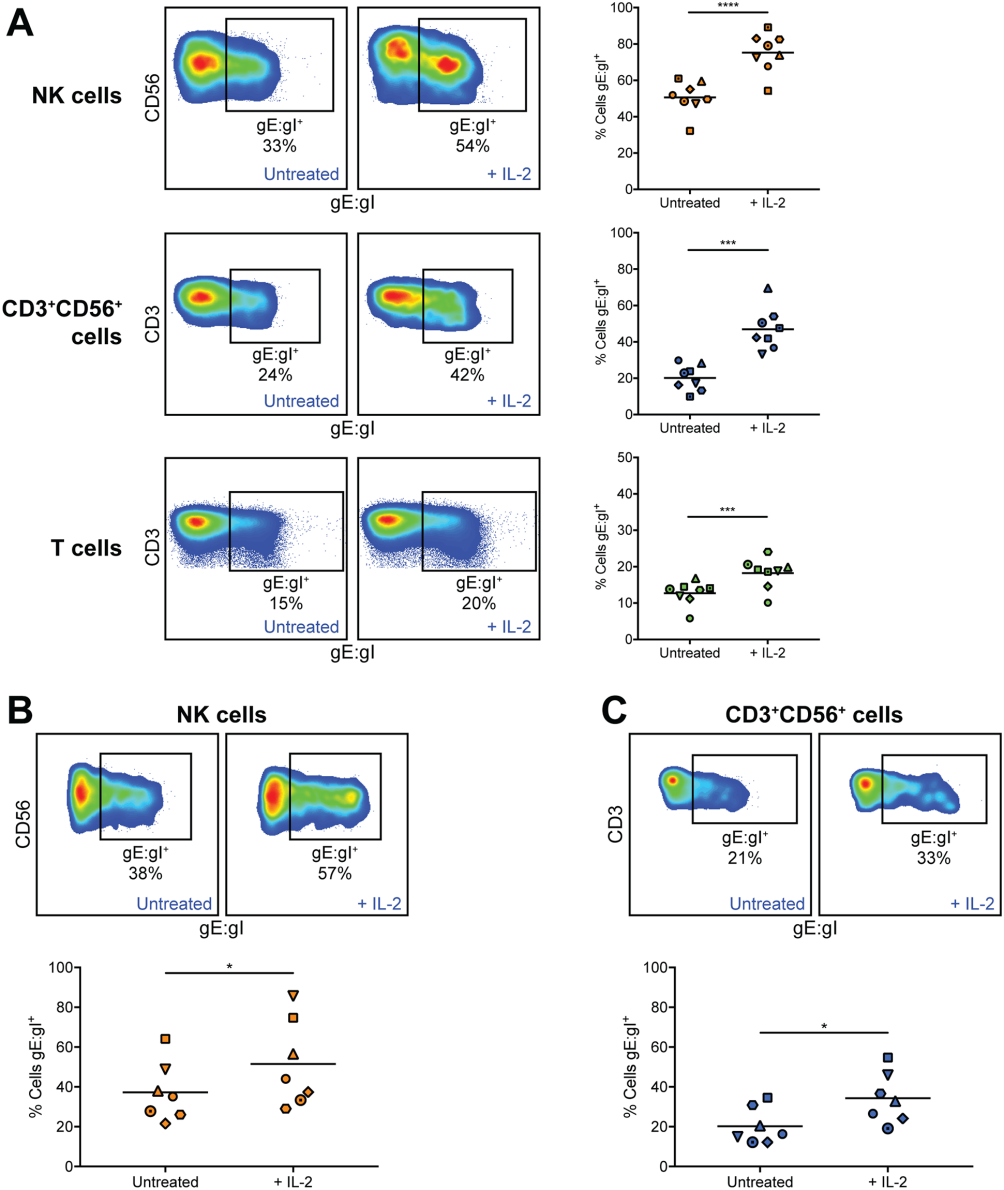
IL-2 stimulation of NK cells, CD3^+^CD56^+^ lymphocytes, and T cells enhances VZV infection. (A) Healthy human donor PBMCs were infected with VZV by cell-associated infection with or without IL-2 (200 U/ml) for 2 days, then analysed by flow cytometry. Plots show surface VZV gE:gI expression from one representative donor and graphs show frequency of live gE:gI^+^ NK cells (CD3^–^CD56^+^) (top panels), CD3^+^CD56^+^ lymphocytes (middle panels), and T cells (CD3^+^CD56^–^) (bottom panels). Symbols represent individual donors consistent across lymphocyte populations, and bars indicate mean (n = 8). ***p < 0.001, ****p < 0.0001 (two-tailed paired t test). (B & C) Healthy human donor CD56^+^-selected lymphocytes were infected with VZV by cell-associated infection with or without IL-2 (200 U/ml) for 2 days, then analysed by flow cytometry. Plots show surface gE:gI expression from one representative donor and graphs show frequency of live gE:gI^+^ NK cells (B) or CD3^+^CD56^+^ lymphocytes (C). Symbols represent individual donors, consistent across (B & C) (n = 7). *p < 0.05 (two-tailed Wilcoxon matched-pairs signed rank test).

We were thus interested in the effect of IL-2 stimulation on VZV infection of lymphocytes. Analysis of PBMCs infected with VZV for 2 days in the presence of 200 U/ml IL-2 or left untreated revealed that IL-2 stimulation yielded significantly higher rates of infection for NK cells (1.5-fold), CD3^+^CD56^+^ lymphocytes (2.6-fold) and T cells (1.5-fold), compared to unstimulated conditions (Fig 2A). To ascertain whether this effect would hold true when lymphocyte populations were infected in isolation, CD56^+^ lymphocytes were isolated and then infected. Fig 2B and 2C shows that the pattern of significantly increased efficiency of infection with IL-2 stimulation was maintained in both NK cells and CD3^+^CD56^+^ lymphocytes (1.4-fold and 1.7-fold increase, respectively) when CD56^+^ cells were isolated and then infected. Furthermore, the data demonstrates that these cell types are permissive to VZV infection when not cultured with total PBMCs.

### NK cells support the full cascade of VZV gene expression and effectively transmit viral infection

**Fig 3:**
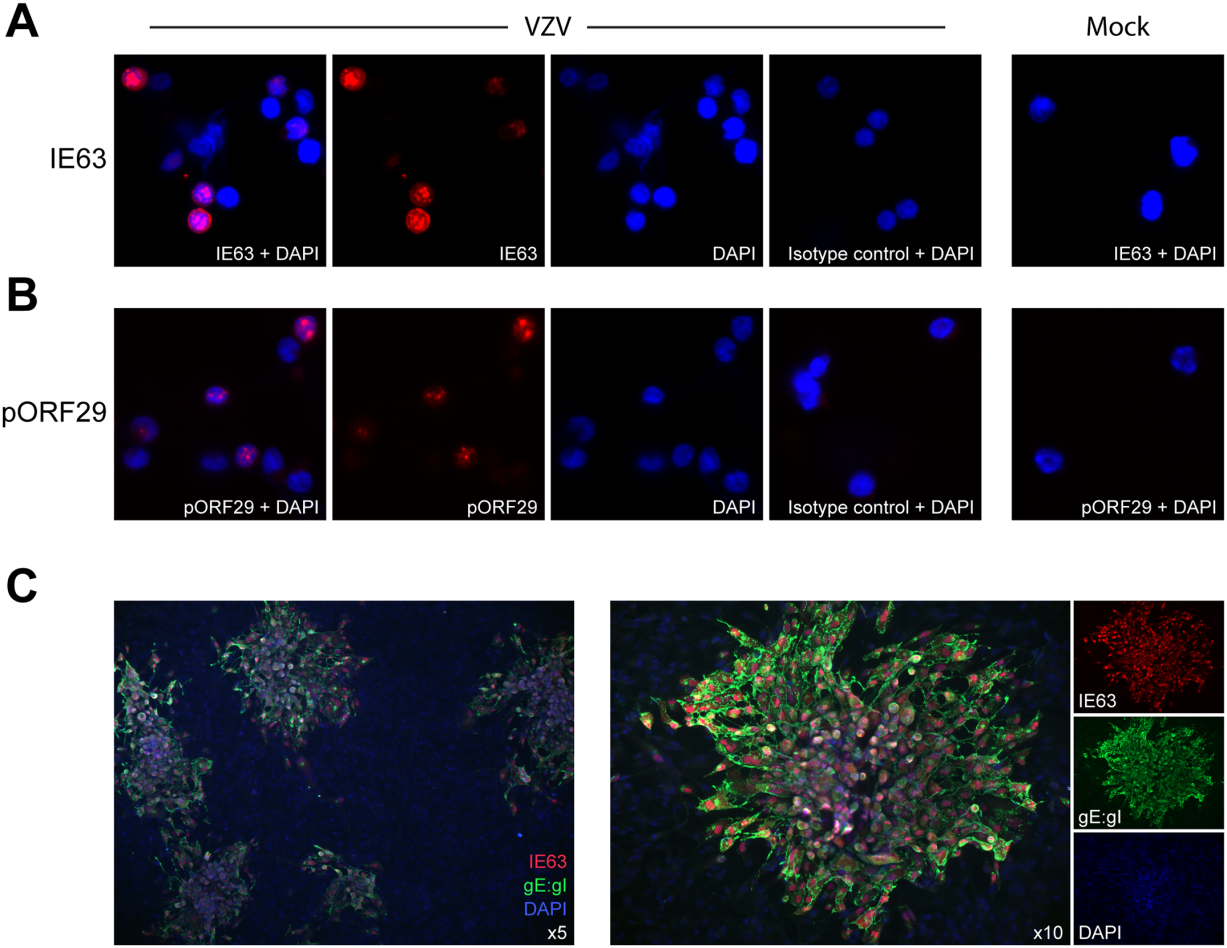
NK cells are productively infected by VZV and support virus transmission. NK cells (CD3^–^CD56^+^) were FACS sorted from healthy human donor CD56^+^-selected lymphocytes following mock or VZV infection for 1 day. (A & B) Staining by IFA of sorted VZV cultured (left panels) or mock cultured (right panels) NK cells for IE63 (A), pORF29 (B) or respective isotype control, with DAPI (n = 3). (C) Sorted VZV cultured NK cells were added to ARPE-19 epithelial cell monolayers. Four days later monolayers were fixed and infectious centres detected with IFA by staining for IE63 and gE:gI, with DAPI. One representative experiment of five is shown.

A unifying characteristic of herpesviruses is that their productive replication cycle occurs through an ordered temporal cascade of gene expression comprising immediate early, early and late genes [50]. Thus far we had demonstrated that NK cells and CD3^+^CD56^+^ lymphocytes expressed late VZV gene product gE:gI on the cell surface and GFP expressed from the viral genome. Given the significantly higher rate of infection of NK cells, we focused on further characterising VZV infection of this lymphocyte population. To establish whether NK cells were able to support the full kinetic cascade of VZV gene expression, CD56^+^-selected lymphocytes were again infected in a cell-associated manner using mock or VZV infected ARPE-19 cells for 1 day, then CD3^–^CD56^+^ NK cells were isolated by FACS sorting before staining for immediate early protein IE63 (Fig 3A) or early protein pORF29 (Fig 3B) for immunofluorescence assay (IFA) analysis. VZV cultured NK cells were found to express IE63 with both cytoplasmic and nuclear localisation (Fig 3A), as previously described [51, 52]. Additionally, the classical punctate nuclear staining of pORF29 [53] was observed in VZV cocultured NK cells (Fig 3B). IE63 and pORF29 could not be detected in mock cultured NK cells, as expected (Fig 3A & 3B; right panels).

We additionally performed quantitative PCR (qPCR) on VZV cultured NK cells to measure viral genome copies. VZV cultured NK cells were isolated away from VZV infected ARPE-19 cells by FACS sorting at 4 hours post infection (hpi). Analysis by qPCR of DNA extracted from VZV cultured NK cells revealed an almost 3-fold increase in viral genome copies from 4 hpi to 12 hpi, which had begun to decrease at 24 hpi but still exceeded viral genomes quantified at 4 hpi (S3 Fig). As qPCR analysis was performed on the total population of NK cells cultured with VZV, the increase in viral genome copies could either indicate increasing genome replication in cells already infected at 4 hpi and/or spread and replication of virus in more cells over time. Both possibilities, however, suggest productive viral replication of VZV in NK cells.

We then sought to clearly establish whether the NK cells were capable of producing *de novo* infectious virus, through use of an infectious centre assay. Using CD56^+^-selected lymphocytes, VZV cultured NK cells (38% gE:gI^+^ on average) were isolated away from the inoculum cells at 1 day post infection by FACS sorting and then added to ARPE-19 epithelial monolayers. After 4 days in culture, plaques in the monolayer were visible by cytopathic effect (CPE) under light microscope, and confirmed by IFA for IE63 and gE:gI expression (Fig 3C), demonstrating that infectious virus was transmitted from NK cells to the epithelial cells. To eliminate the possibility of plaque formation through passive transfer of extracellular virions attached to the surface of NK cells, we also performed the assay with an additional step of citrate buffer washing which is known to inactivate and detach surface-bound virions [54–57]. Isolated NK cells stripped with citrate buffer prior to addition to the epithelial monolayers again resulted in VZV infection and replication as indicated by plaque formation (S4 Fig), suggesting productive transmission of VZV from the infected NK cells to the epithelial cells.

Overall, these results demonstrate that human NK cells can be productively infected with VZV, supporting the full replicative cycle of viral gene expression. Most pertinently, VZV infected NK cells were also able to transmit infectious virus to epithelial cells in culture.

### VZV preferentially infects CD56^dim^ NK cells

**Fig 4:**
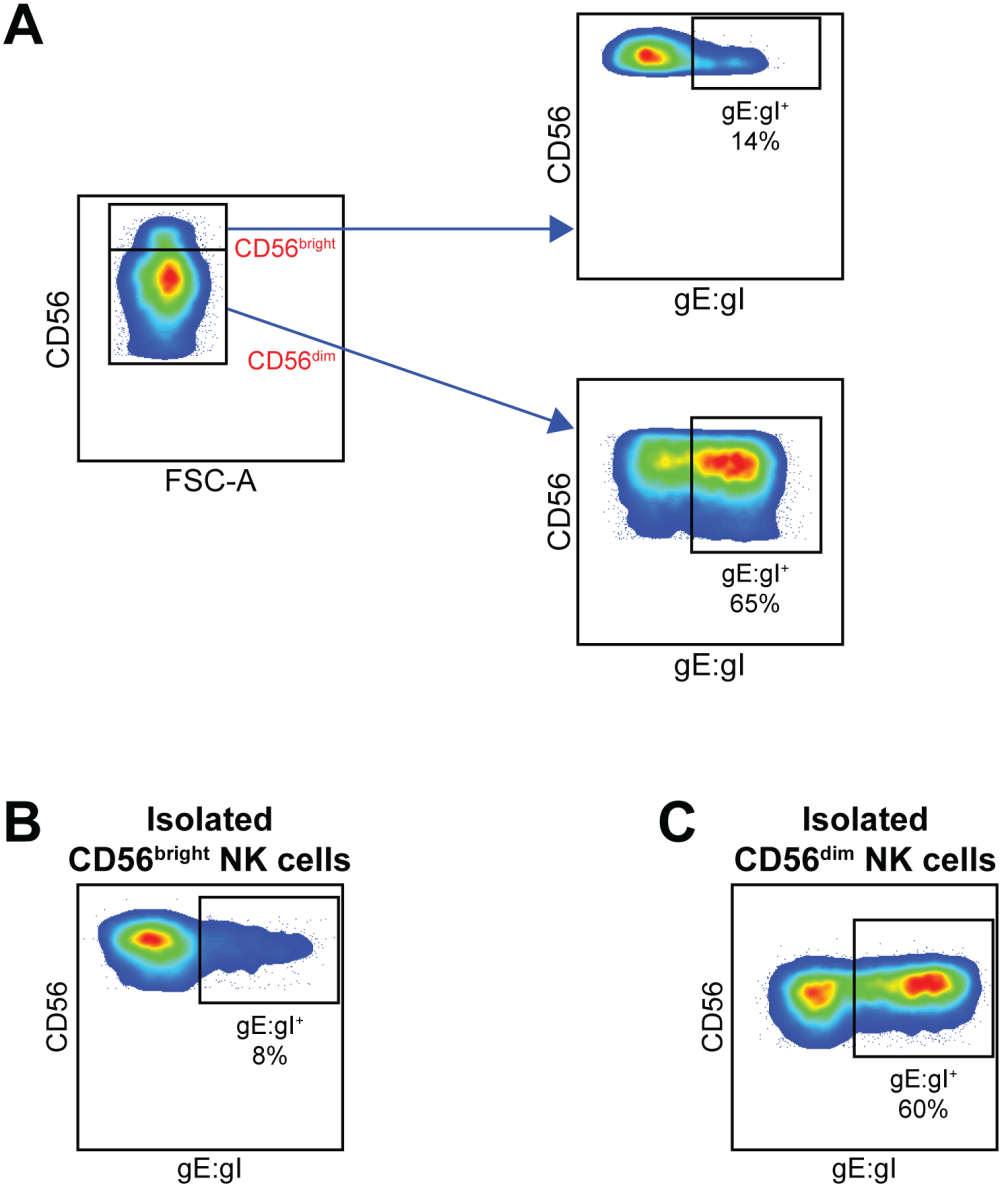
CD56^dim^ NK cells are more effective than CD56^bright^ NK cells at supporting VZV infection. (A) Healthy human donor PBMCs were infected with VZV for 2 days then analysed for infection by flow cytometry. Plots show gating strategy for CD56^bright^ and CD56^dim^ NK cells (CD3^–^CD56^+^) (left panel), with respective surface VZV gE:gI expression (right panels) from one representative donor (n = >7). (B & C) CD3^–^CD56^bright^ (B) and CD3^–^CD56^dim^ (C) NK cells were isolated from healthy human donor PBMCs by FACS sorting and subsequently infected with VZV for 2 days before analysis by flow cytometry. Plots show surface gE:gI expression from one representative donor (n = 2).

In peripheral blood there are two main subsets of NK cells differentiated by their level of expression of CD56. CD56^dim^ NK cells constitute the major subset, while only up to 10% of the circulating population will be CD56^bright^ NK cells. In analysing detection of VZV gE:gI on NK cells infected with VZV it was apparent that infection consistently favoured the CD56^dim^ population (Fig 4A). However, the CD56^bright^ population was not always easy to distinguish due to variability in donors and subtle variation in CD56 expression following culture with VZV. Therefore, to confirm this observation, we sorted CD3^–^CD56^bright^ NK cells and CD3^–^CD56^dim^ NK cells from PBMCs and then inoculated the separate subsets with VZV for 2 days, as described earlier. Detection of gE:gI on isolated NK cell subsets recapitulated the trend seen with total NK cell infection, with 5-fold more CD56^dim^ NK cells infected on average, compared to CD56^bright^ NK cells (Fig 4B and 4C). The data thus suggests a preferential infection of CD56^dim^ NK cells by VZV.

### VZV stimulates loss of CD16 cell-surface expression but drives expression of CD57

CD56^bright^ NK cells are generally regarded as an immature precursor of CD56^dim^ NK cells, although there is still some contention in the field [58]. Given that VZV favoured infection of CD56^dim^ NK cells we asked whether the maturity status of NK cells may influence their permissiveness to VZV infection. The hallmark of NK cell maturity is expression of CD57, which is also correlated with increased expression of CD16, KIRs, and LIR-1, and a loss of NKG2A expression [35, 36].

**Fig 5:**
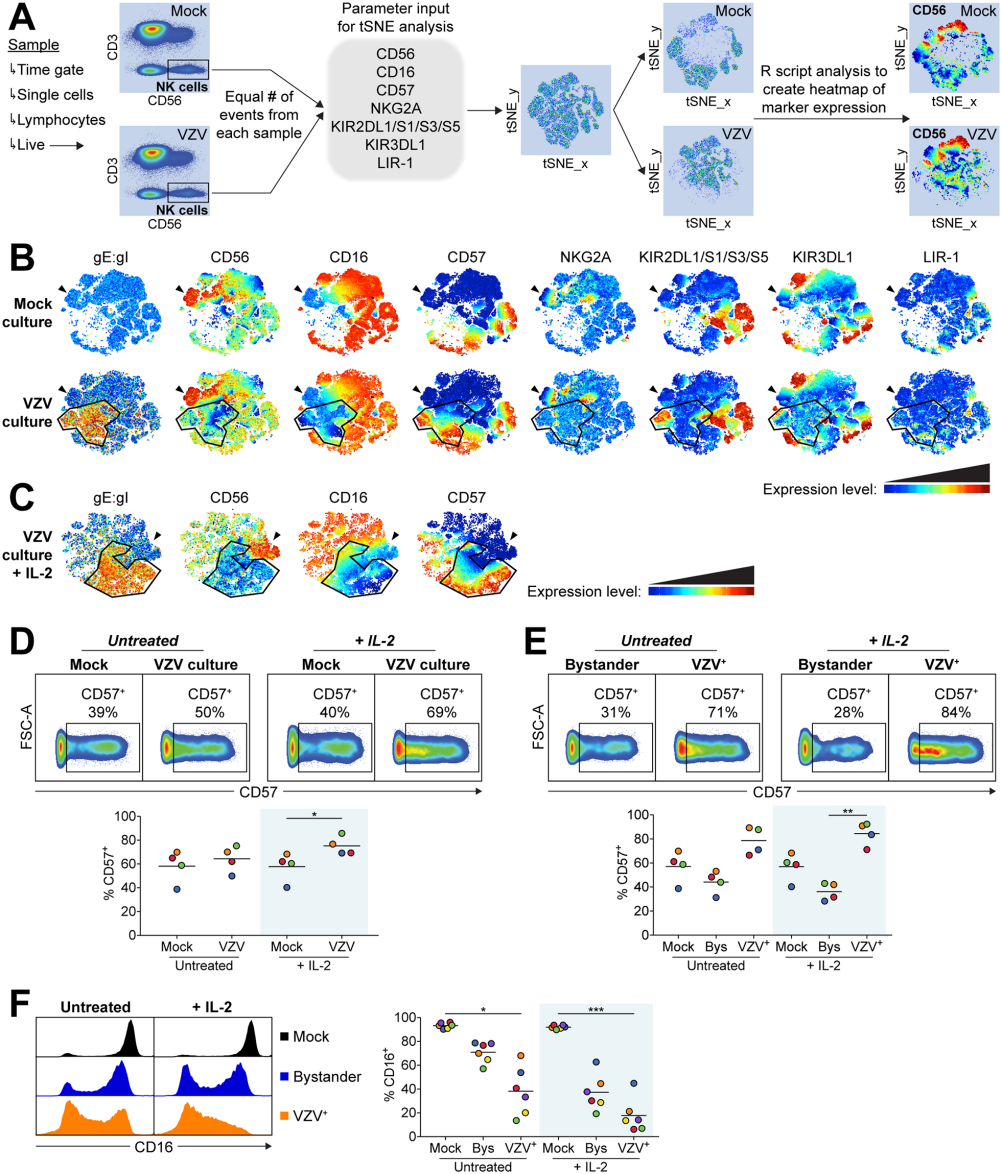
NK cell markers associated with maturity influence VZV infection of NK cells and are modulated by VZV. Healthy human donor PBMCs were mock or VZV infected with or without IL-2 (200 U/ml) for 2 days then analysed by flow cytometry. (A) Diagram describes gating strategy and tSNE analysis workflow for samples shown in (B & C). (B & C) tSNE plots show marker expression levels for single parameters on individual cells in the tSNE map for mock and VZV cultured NK cells after 2 days, either untreated (B) or in the presence of IL-2 (C). Arrowheads indicate the CD56^bright^ NK cell subset, and the outlined population indicates the localisation of VZV^+^ NK cells. One representative experiment of three is shown. (D & E) Plots show CD57 expression between mock and VZV cultured NK cells (D) and between bystander and VZV^+^ NK cells (E), from one representative donor. Graphs show respective frequencies of CD57^+^ NK cells when untreated or with IL-2 (shaded) for four donors. Bars indicate mean. (F) Histograms show CD16 expression for mock, bystander and VZV^+^ NK cells from one representative donor. Graph shows frequency of CD16^+^ NK cells when untreated or with IL-2 (shaded) for six donors. Bars indicate mean. *p < 0.05, **p < 0.01, ***p < 0.001 (Friedman test with Dunn’s multiple comparisons test).

To evaluate the influence of NK cell maturity on VZV infection we used a non-linear dimensionality reduction algorithm approach to interpret high dimensional flow cytometry data. As shown in Fig 5A, the t-distributed stochastic neighbour embedding (tSNE) algorithm was configured to distribute data combined from mock and VZV samples according to expression of the NK cell maturity markers CD56, CD16, CD57, NKG2A, KIR2DL1/S1/S3/S5, KIR3DL1 and LIR-1. VZV gE:gI was not included as a parameter for tSNE analysis so that populations would be defined only by their expression of maturity markers. This allowed subsequent visualisation within the tSNE map of where gE:gI expression was localised, identifying the VZV infected (VZV^+^) NK cells.

Analysis with the tSNE algorithm displayed expected correlations of marker expression, such as NKG2A expression being predominantly limited to the CD56^bright^ NK cell population (Fig 5B). It was also apparent that while CD56^dim^CD57^+^ NK cells clustered together, KIR and LIR-1 expression did not localise to one discrete population. In comparing mock to VZV culture, a distinct VZV^+^ population was apparent which was not present in the mock culture, illustrating that VZV infection has modulated expression of maturity markers to an extent substantial enough to distinguish the cells. This profile was consistent across the 3 donors analysed. As shown in Fig 5B, VZV did not substantially change expression, or co-localise with, KIRs, LIR-1 or NKG2A. In contrast, the defining changes in the VZV^+^ population were lower CD56 and CD16 expression, as well as considerable co-localisation with CD57 expression. VZV infection performed in the presence of 200 U/ml IL-2 potentiated these trends (Fig 5C).

Closer analysis revealed the frequency of CD57^+^ NK cells increased with VZV culture, which was significantly enhanced when stimulated with IL-2 (Fig 5D). While 2 days of IL-2 activation was not sufficient for mock NK cells to develop increased CD57 expression, we observed that IL-2 stimulation concurrent with VZV culture markedly enhanced NK cell expression of CD57 compared to untreated VZV culture (Fig 5D). Delineating analysis of VZV cultured NK cells into VZV^+^ and bystander (gE:gI^–^) revealed a higher frequency of CD57^+^ NK cells in the VZV^+^ population compared to bystander or mock NK cells (Fig 5E). Additionally, comparison of CD16 expression between mock, bystander and VZV^+^ NK cells demonstrated a reduced frequency of CD16^+^ NK cells in the bystander population, which was further diminished to a significant level in the VZV^+^ NK cells (Fig 5F). The frequency of the CD16^+^ populations were significantly further reduced in the presence of IL-2. Furthermore, of the cells that were CD16^+^, a clear decrease in CD16 MFI was apparent in bystander and VZV^+^ NK cells (Fig 5F). These results demonstrate a striking reduction in CD16 expression induced by culture and infection with VZV, leading NK cells to either completely lose expression of CD16, or experience decreased intensity of expression.

**Fig 6:**
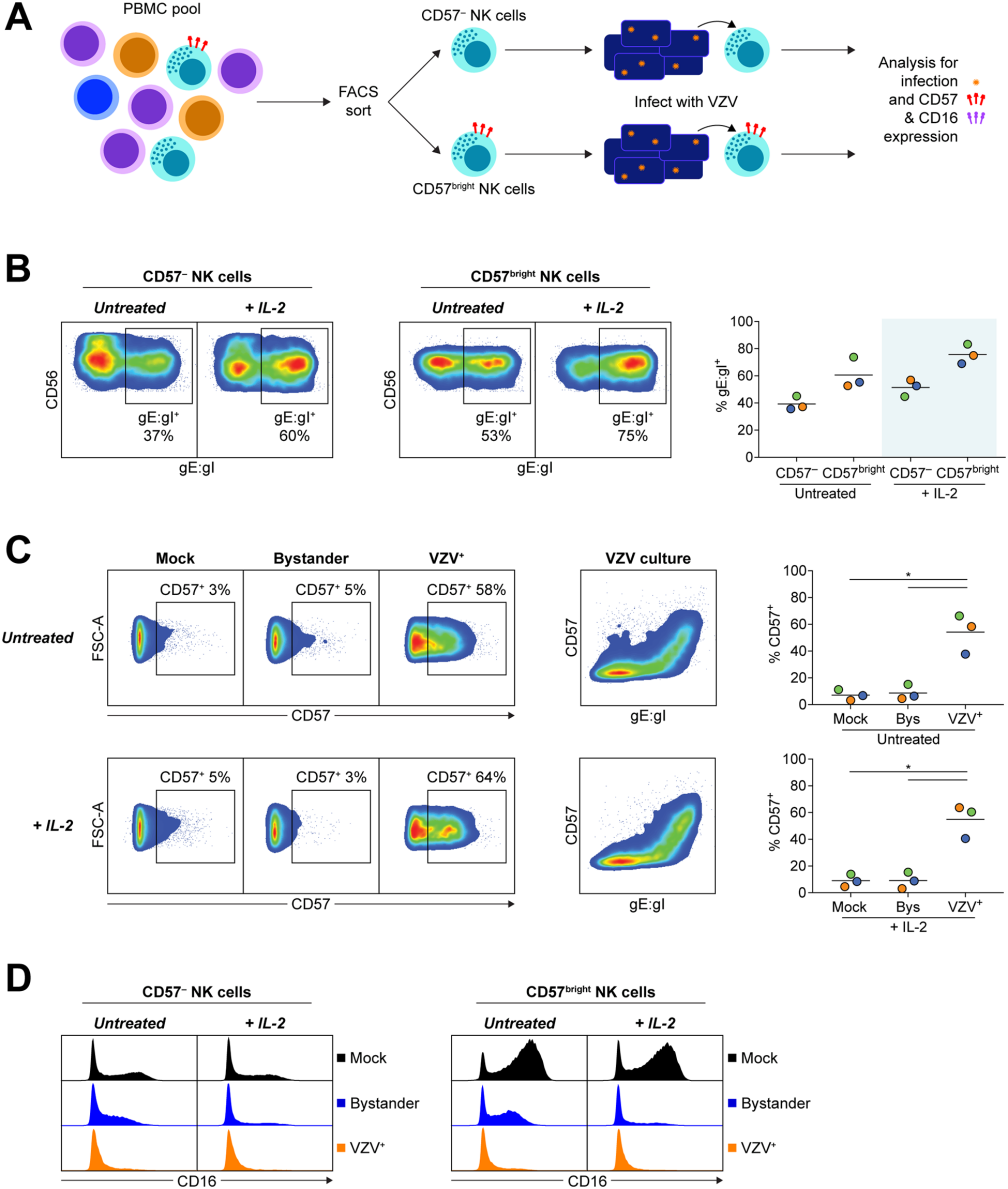
VZV infects both CD57^–^ and CD57^bright^ NK cells and drives CD57 expression. CD3^–^CD56^+^CD57^–^ NK cells and CD3^–^CD56^+^CD57^bright^ NK cells were isolated from healthy human donor PBMCs by FACS sorting and subsequently mock or VZV infected with or without IL-2 (200 U/ml) for 2 days before analysis by flow cytometry. (A) Diagram describes experimental design of isolating NK cells on CD57 expression, then infecting, and subsequently analysing for infection and phenotype changes. (B) Plots show surface VZV gE:gI expression between subsets from one representative donor. Graph shows frequency of VZV^+^ NK cell subsets when untreated or with IL-2 (shaded) for three donors. Bars indicate mean. (C) Plots show subsequent CD57 expression between mock, bystander and VZV^+^ CD57^–^ NK cells (left panels) and CD57 versus gE:gI expression for VZV cultured CD57^–^ NK cells (middle panels), from one representative donor. Graphs show frequency of CD57 expression on mock, bystander and VZV^+^ CD57^–^ NK cells for three donors. Bars indicate mean. *p < 0.05 (two-tailed paired t test). (D) Histograms show CD16 expression for mock, bystander and VZV^+^ CD57^–^ NK cells (left panel) and CD57^bright^ NK cells (right panel) for one representative donor (n = 3).

The reduced frequency of CD57^+^ NK cells in the bystander population compared to VZV^+^ or mock NK cells (Fig 5E) could be explained in several ways. One possibility is that the bystander cells have downregulated CD57 expression. Alternatively, the data could be interpreted as preferential infection of CD57^+^ NK cells which would deplete the frequency of CD57^+^ NK cells in the bystander population. To distinguish between these possibilities, we isolated CD3^–^CD56^+^CD57^–^ NK cells and CD3^–^CD56^+^CD57^bright^ NK cells by FACS sorting PBMCs and then cultured these cells with VZV or mock inoculum for 2 days in the presence or absence of 200 U/ml IL-2 (Fig 6A). Both CD57^–^ and CD57^bright^ NK cells were found to support VZV infection (39% ± 3% gE:gI^+^ and 61% ± 7% gE:gI^+^, respectively). CD57^bright^ NK cells were more frequently infected, however, with a 1.5-fold increased expression of gE:gI compared to the CD57^–^ NK cell population (Fig 6B). Both subsets also experienced a 1.3-fold increase in permissiveness to VZV with 200 U/ml IL-2 stimulation.

Most striking from the infection of CD57^–^ NK cells with VZV was the observation that VZV drove significant expression of CD57 (Fig 6C). While only a small percentage of mock and bystander CD57^–^ NK cells acquired CD57 expression in untreated conditions (7% ± 2% and 9% ± 3%, respectively), 54% ± 8% of VZV^+^ CD57^–^ NK cells became CD57^+^. In this setting, we did not observe enhanced acquisition of CD57 with IL-2 stimulation for mock or VZV cultured CD57^–^ NK cells compared to untreated. Additionally, culture of CD57^–^ and CD57^bright^ subsets with VZV revealed that both subsets had reduced CD16 expression in the bystander and VZV^+^ populations compared to mock culture (Fig 6D), supporting the observations in total NK cells (Fig 5F). Combined, these results present a complex picture of VZV possessing some preference for infection of more mature NK cells, but also modulating expression of maturation markers on the surface of NK cells.

### VZV increases expression of skin-homing chemokine receptors

**Fig 7:**
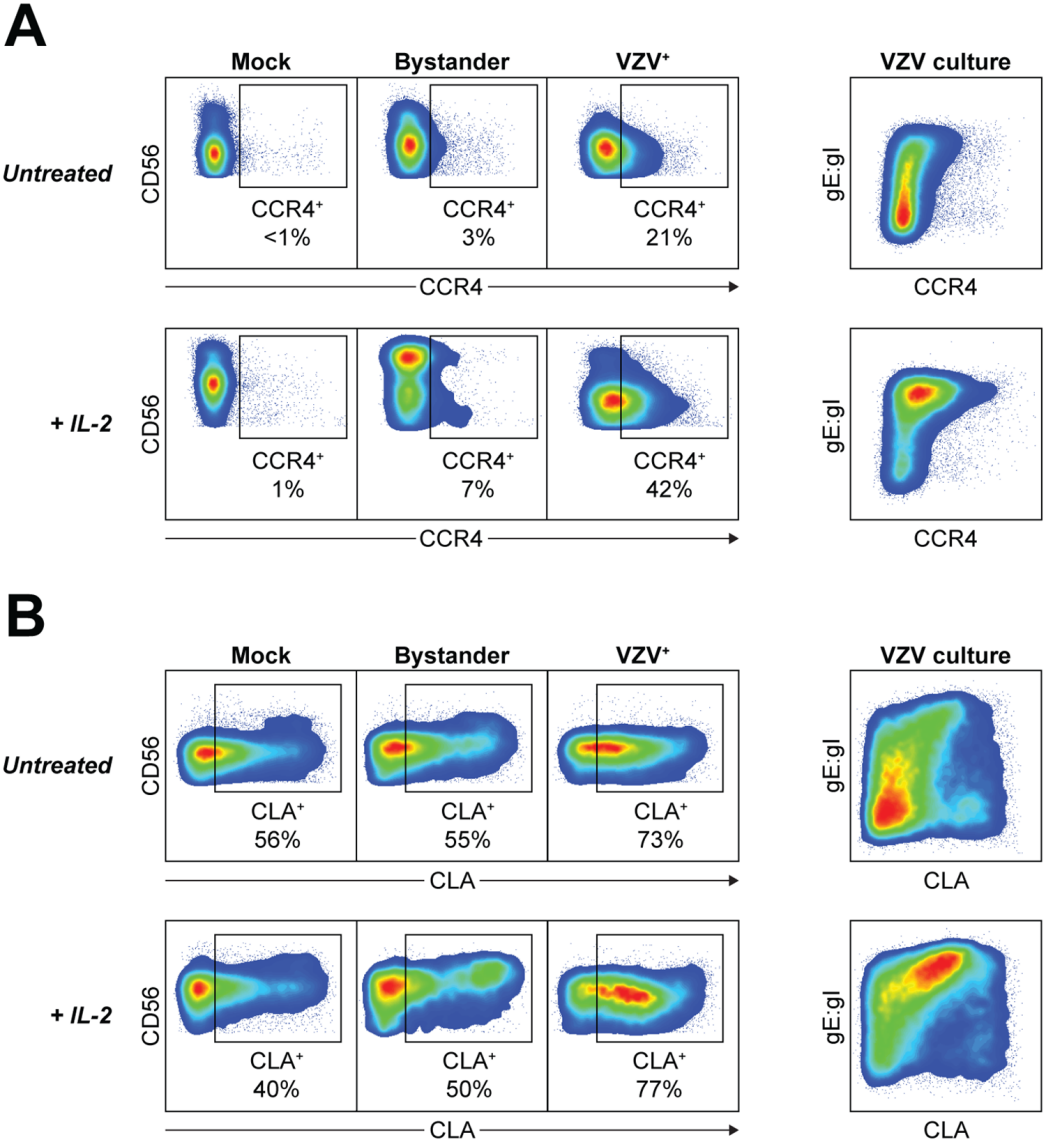
VZV upregulates expression of skin-homing chemokine receptors on NK cells. Healthy human donor PBMCs were mock or VZV infected with or without IL-2 (200 U/ml) for 2 days then analysed by flow cytometry. (Left panels) Representative plots show CCR4 (A) or CLA (B) expression against CD56 expression for mock, bystander and VZV^+^ NK cells. (Right panels) Representative plots show CCR4 (A) or CLA (B) expression versus VZV gE:gI expression for VZV cultured NK cells. Data are representative of five donors.

It has been previously reported that VZV infection of T cells leads to remodelling of the cell surface phenotype including upregulation of skin-homing markers CCR4 and cutaneous lymphocyte antigen (CLA) [59]. We thus sought to determine if NK cells infected with VZV would also change in their expression of chemokine receptors involved in migration to the skin. Comparison between mock, bystander and VZV^+^ NK cells after 2 days coculture revealed that VZV infection induced upregulated expression of both CCR4 and CLA chemokine receptors (Fig 7). Limited expression of CCR4 was detected on mock NK cells, as well as bystander NK cells, which was in striking contrast to VZV^+^ NK cells where CCR4 expression was observed on 26% ± 4% of cells (Fig 7A). Stimulation of NK cells with 200 U/ml IL-2 led to further enhancement of CCR4 expression on VZV^+^ NK cells, with 40% ± 6% CCR4^+^. VZV infection also induced upregulation of CLA expression, above the high constitutive frequency of CLA^+^ NK cells observed in the mock culture, and maintained on bystander NK cells (Fig 7B). Overall, it is apparent that VZV manipulates the expression of chemokine receptors on infected NK cells to promote a skin-homing phenotype.

## Discussion

NK cells are an essential component of the early host response to infection, and have been implicated in responding to VZV infection during both varicella and herpes zoster [60–63]. Considering this, it is surprising that characterisation of how VZV interacts with NK cells has mostly not been pursued. Particularly in the context of establishing infection where there is an indispensable requirement for VZV to infect immune cells and hijack their migratory function to disseminate virus, a role for NK cells in this process has not been investigated to date. We show here for the first time that human peripheral blood NK cells support productive VZV infection, and that VZV actively manipulates the NK cell phenotype which may influence the course of host infection. These findings reveal a dynamic interplay between VZV and the NK cell immune response, and propose a new avenue for consideration in the study of pathogenesis and immune evasion by VZV.

Our analysis of cell-associated VZV infection of healthy human PBMC indicated novel infection of NK cells and CD3^+^CD56^+^ lymphocytes. CD3^+^ T cells that express CD56 are a heterogeneous population which includes type I and type II NKT cells, and subsets of γδ T cells and mucosal-associated invariant T (MAIT) cells [64]. It would be interesting to characterise further here the cell types permissive to VZV given the ability for these lymphocyte populations to migrate to distinct sites around the body. In comparison, considering that NK cell infection has not been previously investigated, it was remarkable to see up to 65% of live NK cells becoming infected with VZV– significantly more than T cells or CD3^+^CD56^+^ lymphocytes. Increasing appreciation for the breadth of diversity in the NK cell pool [65] prompted investigation into whether VZV may be preferentially infecting a subset of NK cells. Indeed, we observed enhanced infection of CD56^dim^ NK cells, as well as CD57^bright^ NK cells, both of which compose mature NK cell populations. Analysis with tSNE showed VZV infection through a range of mature NK cell phenotypes (both KIR^–^CD57^+^CD56^dim^ NK cells and the more differentiated KIR^+^CD57^+^CD56^dim^ NK cells) that fall within the non-linear differentiation pathway of NK cell maturation [35]. Infection of mature NK cell subsets parallels the predilection of VZV to infect tonsillar memory T cells– a mature T cell population [4]. The preference of VZV to infect mature lymphocytes may play into the increased severity of varicella experienced in adults [66], as a larger pool of lymphocytes capable of supporting infection would enhance dissemination of virus. Furthermore, as CD56^dim^ NK cells predominantly circulate in the blood, preferential infection of this subset would align with a role for VZV infection of NK cells during VZV viremia.

In conjunction with preferential infection of mature NK cell subsets, VZV infection of purified CD57^–^ NK cells led to a potent upregulation of maturity marker CD57 on the cell surface. Although expression of CD57 is used to define terminally-differentiated mature NK cells, it is not well understood how CD57 expression is acquired. It has been demonstrated that moderate CD57 acquisition can be driven by treatment with IL-2 [36], IL-15 [35] and a glycogen synthase kinase (GSK) 3 inhibitor [67]. However, while these studies treated NK cells for 5–7 days to induce CD57 expression, we observed marked acquisition of CD57 within 2 days of VZV infection. Although upregulation of CD57 and expansion of CD57^+^ NK cells occurs in response to pathogen exposure, we believe this is the first evidence of a direct viral infection of NK cells driving CD57 expression. Despite the identification of CD57 as a marker of mature NK cells with refined function [36], it is currently unclear whether CD57 mediates these changes or merely denotes NK cells of a mature phenotype. Furthermore, as a carbohydrate epitope, CD57 is not independently expressed on the cell surface but rather is bound to surface glycoproteins. As previously noted [68], while CD57 expression has been reported on the IL-6 receptor of lymphocytes [69], which molecules CD57 is predominantly expressed on for NK cells remains to be determined. In our study, it is possible that CD57 is being expressed on a surface viral glycoprotein, explaining the marked CD57 expression acquired by purified CD57^–^ NK cells when infected with VZV. Alternatively, the process of viral replication may stimulate the activity or enhanced expression of B3GAT1, the key enzyme in the biosynthesis of CD57. Further research into how CD57 expression is acquired by mature NK cells is warranted, and our findings may help in delineating this process.

Analysis by tSNE of NK cell maturity markers detected by flow cytometry revealed substantial remodelling of the NK cell phenotype by VZV infection. The plot produced by tSNE analysis arranges cells based on their similarity of expression of the input markers, and thus the distinct cluster of VZV^+^ NK cells absent in the mock culture indicates altered expression unique to VZV infection. In addition to the upregulation and co-localisation of CD57 expression with VZV gE:gI expression, the other clear observation was a dramatic loss of CD16 on the cell surface– an effect that occurred with both infected and bystander NK cells. This finding is particularly interesting given that CD57^+^ NK cells are reported to be highly responsive to ADCC stimulation through CD16 [36]. As activation of NK cells has been demonstrated to instigate metalloprotease-mediated cleavage of CD16 from the cell surface [70, 71], it is possible that the decreased expression of CD16 observed with VZV culture could be a product of viral sensing through NK cell pathogen recognition receptors. For HCMV and influenza, virally infected targets cultured with seropositive donor sera stimulate CD16-dependent expansion of NK cells and IFN-γ production [72], thus downregulation of CD16 may be a tactic through which VZV circumvents a critical immune pathway of viral control.

In many of our experiments we examined the outcome on NK cells cocultured with VZV in absence or presence of IL-2. As one of the main cytokines that act on NK cells, IL-2 stimulates global activation of NK cells, enhancing cytotoxicity, modulating surface receptors, and promoting survival [73]. We observed that stimulation of NK cells with IL-2 during VZV infection increased the frequency of infected NK cells detected. In order to maintain efficient viral replication, it has been demonstrated that VZV triggers phosphorylation of signal transducer and activator of transcription 3 (STAT3) [74]. As IL-2 signals through phosphorylation of Janus kinase 1 (Jak1) and Jak3 with subsequent activation of STAT3 and STAT5 [75], it is possible that IL-2 stimulation of NK cells augments viral replication, yielding an increased frequency of infected NK cells. Alternatively, as IL-2 has been shown to have a maturing effect on NK cells [24], treatment with IL-2 may allow refractive NK cells to become supportive of VZV infection. Furthermore, we observed that changes in cell-surface phenotypes induced by VZV infection were potentiated with IL-2 stimulation. VZV-induced upregulation of CD57, loss of cell-surface CD16, and increased expression of homing receptors were all enhanced with IL-2 stimulation. Given the sensitivity of NK cells to cytokine-mediated changes in function, phenotype, proliferation and survival [76], it would be illuminating to examine other cytokines involved in viral infection and investigate their influence on the effects exerted by VZV on NK cells. Our findings with IL-2 highlight the importance of considering the microenvironment in which viral-immune cell interactions take place, especially as IL-2 production by activated T cells and DCs is abundant during infection.

NK cells are readily able to migrate around the body to sites of inflammation, including trafficking to inflamed skin, which has been reported to occur in herpes zoster [77, 78]. While functional migration requires a combination of changes in chemokine receptor expression, key markers associated with skin homing include CCR4 and CLA [79]. In accordance with the immunosurveillance role of NK cells, we found CLA to be constitutively expressed on a distinct portion of NK cells, and following VZV infection, the frequency of CLA^+^ NK cells was increased. Notably, while CCR4 was mostly absent on the surface of mock NK cells, we observed a substantial increase in CCR4 expression driven by infection with VZV. This remodelling of chemokine receptor expression to induce a skin-homing phenotype is analogous to previous reports for T cells [59], and thus may indicate a pan-lymphocyte mechanism employed by VZV. Virally induced changes in chemokine receptor expression would allow VZV to hijack lymphocyte trafficking to promote viral dissemination to the skin where highly infectious lesions can develop. In fact, in a report of severe varicella in immunocompetent children, NK cells and primed T cells were significantly reduced in frequency in circulation during the early phase of infection, which was hypothesised to be a result of migration to peripheral sites such as the skin [80].

Case reports of severe varicella infection in patients with NK cell deficiency indicate that NK cells are critical in controlling VZV infection [81]. Our data demonstrate however that VZV is not passive in its interaction with NK cells. We demonstrate that VZV productively infects NK cells and remodels the surface phenotype in amplifying expression of skin-homing chemokine receptors. We propose that along with the established roles for T cells and DCs, that NK cells may also be active players in VZV pathogenesis and could contribute to the dissemination of virus. We have additionally demonstrated substantial changes in NK cell maturity markers driven by VZV infection. How lifelong pathogens, like VZV, interact with NK cells is significant in comprehending the context in which these immune cells exist in the human host, as well as progressing our understanding of immune interactions during VZV infection.

## Materials and methods

### Immune cell isolation

Healthy human donor buffy coats were obtained through the Australian Red Cross Blood Service from which PBMCs were isolated by density gradient centrifugation with Ficoll-Paque PLUS (GE Healthcare) and resuspended in complete RPMI medium (RPMI 1640 with L-glutamine [Lonza] supplemented with 10% human serum [Sigma-Aldrich]) for subsequent experiments. For some experiments, where specified, CD56^+^ lymphocytes were isolated from PBMC by MACS positive selection using CD56 MicroBeads, according to manufacturer’s protocol (Miltenyi Biotech). CD56^+^-selected lymphocytes were then resuspended in complete RPMI medium for subsequent experiments. For FACS sorting of lymphocyte populations and subsets, PBMCs were stained with fluorochrome-conjugated antibodies in FACS buffer on a rocking platform, and sorted to >95% purity using a FACSAria IIu or BD Influx (both BD Biosciences). Cells were kept on ice during the sorting process.

### Cell culture and viruses

ARPE-19 epithelial cells (ATCC) were cultured in complete DMEM medium (DMEM with 4.5 g/L glucose and L-glutamine [Lonza] supplemented with 10% foetal calf serum [FCS] and penicillin streptomycin). VZV-S and rOka-ORF10-GFP (VZV-GFP), which expresses GFP as a fusion protein with ORF10 [82], (both kindly provided by A Arvin, Stanford University), were propagated in ARPE-19 cells in complete DMEM medium. For vOka infections, VARIVAX (varicella virus vaccine Oka/Merck Strain) (MSD) was inoculated onto ARPE-19 cells and passaged in complete DMEM medium. For cell-free VARIVAX infections, the vaccine preparation was used as provided.

### Viral infection of immune cells

Infection cocultures were performed with uninfected (for mock) or VZV infected ARPE-19 cells at a CPE of 2+ to 3+ (approximately 50–75% of cells showing altered morphology). ARPE-19 cells were trypsinised, washed and resuspended in complete RPMI medium for addition with lymphocytes at a ratio of 1:2–1:5 ARPE-19 to lymphocytes. For PBMCs, infections were conducted in 12-well plates using 1–2 x 10^6^ PBMCs in 1 ml of complete RPMI medium. For CD56^+^-selected lymphocytes and FACS sorted subsets, infections were conducted in 24-well plates using 2–4 x 10^5^ lymphocytes in 600 μl of complete RPMI medium. Cell-free infections were performed at an MOI of 0.001 using 1 x 10^6^ PBMCs in 1 ml of complete RPMI medium in 12-well plates. The next day PBMCs were washed in PBS to remove the cell-free virus preparation, and then PBMCs were returned to culture. For both infection methods, cells were spinoculated in tissue culture plates by centrifuging plates at 150 g for 15 mins at room temperature. Plates were then cultured at 37°C with 5% CO_2_ for 2 days, unless otherwise specified. In some experiments lymphocytes were stimulated with 200 U/ml human IL-2 IS (“Improved Sequence”) (Miltenyi Biotec) for the duration of coculture.

### Antibodies

For flow cytometry and FACS sorting experiments, fluorochrome-conjugated antibodies against the following antigens were used: CD56 (clone NCAM16.2; conjugated to BV605) (B159; APC), CD3 (SK7; BUV395) (HIT3a; PE), CD57 (NK-1; BV421), CD16 (3G8; BV510 and PE/Cy7), KIR3DL1 (DX9; BV711) (all BD Biosciences), CD57 (HCD57; PE), LIR-1 (GHI/75; AF647), CCR4 (L291H4; BV421), CLA (HECA-452; AF647) (all BioLegend), KIR2DL1/S1/S3/S5 (HP-MA4; PerCP/Cy5.5) (Abcam), NKG2A (131411; PE) (R&D Systems), and VZV gE:gI (SG1-1; conjugated in-house to DyLight 488) (Meridian Life Science). Matched isotype control antibodies were also used where appropriate. For IFA experiments, unconjugated antibodies against the following antigens were used: gE:gI (clone SG1-1; Meridian Life Science), IE63 (rabbit polyclonal) and pORF29 (rabbit polyclonal) (both kindly provided by P Kinchington, University of Pittsburgh). Fluorochrome-conjugated secondary donkey antibodies were purchased from Life Technologies and Invitrogen (both Thermo Fisher Scientific).

### Flow cytometry

Cells were collected and first stained with Zombie NIR fixable viability dye (BioLegend), according to manufacturer’s protocol. Cells were then resuspended in FACS buffer (PBS supplemented with 1% FCS and 10 mM EDTA) with addition of antibodies at 4°C for at least 30 mins. Thereafter cells were fixed in 1% formaldehyde (Cytofix; BD Biosciences) at 4°C for at least 15 mins. Cells were acquired on an LSR-II cytometer (BD Biosciences).

### Flow cytometry data analysis

Data were analysed with FlowJo software (versions 10.0.7 and 10.2; Tree Star). All data depicted are gated on live cells, as determined by Zombie NIR fixable viability dye staining.

To allow for the visualisation of complex flow cytometry data, we used the Barnes-Hut implementation of t-distributed stochastic nearest neighbour embedding (tSNE), a non-linear dimensionality reduction approach [83, 84]. Using tSNE, individual cells are mapped onto a 2D plot where cells with similar properties in high-dimensional space are grouped together. For analysis, raw flow cytometry data was compensated in FlowJo and the NK cell population was defined by the following gating strategy. A time gate was used to remove aberrant events, single cells were gated on forward scatter height versus forward scatter area, lymphocytes were gated by characteristic forward scatter versus side scatter attributes, live cells were gated by excluding Zombie dye positive cells, and finally NK cells were defined as CD3^–^CD56^+^. NK cell populations were then exported from each sample and re-imported into FlowJo to randomly down-sample to an equal number of cells per sample using the ‘DownSample’ plugin in FlowJo. These samples were concatenated into a single .fcs file, then re-imported into FlowJo where tSNE was performed using the ‘TSne’ plugin. tSNE maps were generated using data from the following parameters: CD56, CD16, CD57, NKG2A, KIR2DL1/S1/S3/S5, KIR3DL1 and LIR-1; under the following tSNE settings: Iteration 1000, Perplexity 30, Eta 200, Theta 0.5. As the samples were concatenated, following tSNE analysis samples were separated by gating on each sample and exporting individual populations as .fcs files for subsequent analysis in FlowJo. To create coloured tSNE plots, the samples were exported from FlowJo as CSV–Channel values so that bi-exponential expression can be visualised on a linear axis and linear colour gradient. A custom script in R [85] was then used with packages ggplot2, colourRamps, ggthemes and scales, which coloured individual cells in the tSNE map by the level of expression of various markers.

### Immunofluorescence assay (IFA)

Indirect IFA was performed on cell spots of FACS sorted VZV or mock cultured NK cells (CD3^–^CD56^+^). Cell spots were air dried on glass slides and fixed with 4% formaldehyde (Cytofix; BD Biosciences) at room temperature. Subsequently, cell spots were permeabilised with 0.1% Triton X-100 (Sigma-Aldrich) for 10 mins at room temperature. Cell spots were blocked with 20% donkey serum, and then stained against IE63 (1:500), pORF29 (1:750), or respective isotype control, for 1 hr at 37°C. Thereafter cell spots were stained with fluorochrome-conjugated secondary antibodies, and finally mounted with ProLong Gold Antifade Mountant with DAPI (Thermo Fisher Scientific). Imaging was performed with a ZEISS Axio Imager.M2 upright microscope with a ZEISS AxioCam HRm digital monochrome CCD camera for fluorescence imaging and ZEISS ApoTome.2 (Carl Zeiss Microscopy).

### Infectious centre assay

CD56^+^-selected lymphocytes were cocultured with VZV infected ARPE-19s for 1 day, as detailed above. Cells were harvested, stained for FACS sorting, and CD3^–^CD56^+^ NK cells isolated. In some experiments, where specified, isolated NK cells were washed in room temperature citrate buffer (40 mM C_6_H_5_O_7_Na_3_, 135 mM NaCl, 10 mM KCl [pH 3]) for 2 mins then washed in PBS. In duplicate, 10^4^ NK cells were then resuspended in complete DMEM medium and added to ARPE-19 monolayers pre-seeded at 7.5 x 10^4^ cells on coverslips in 24-well plates. Plates were spinoculated by centrifuging at 150 g for 15 mins at room temperature, and then incubated at 37°C with 5% CO_2_ for 4 days by which time substantial CPE was observed. Plaques were observed by eye under an inverted light microscope (ZEISS Axio Scope.A1 FL LED; Carl Zeiss Microscopy) before monolayers were fixed in 4% formaldehyde. Viral antigens were subsequently detected by indirect IFA as described above, staining against IE63 (1:500) and gE:gI (1:600), or respective isotype controls. Plaques were visualised with a ZEISS Axio Imager.M2 microscope, detailed above.

### Quantification of viral genome copies

PBMCs were cocultured with VZV infected ARPE-19s for 4 hours, as detailed above. Cells were harvested, stained for FACS sorting, and CD3^–^CD56^+^ NK cells isolated. Throughout the staining and sorting process cells were kept at 4°C or on ice. Following the NK cell isolation, 1 x 10^5^ cells were harvested by washing in PBS and the cell pellet frozen at –80°C. The remaining NK cells were cultured in complete RPMI medium at 37°C with 5% CO_2_ in a 96-well plate, and harvested at the specified time points. Subsequently, DNA was extracted with the QIAamp DNA Mini Kit (QIAGEN), following the protocol for cultured cells. DNA was amplified by qPCR (LightCycler 480 II, Roche) using 2X Brilliant II SYBR Green QPCR Master Mix (Agilent Technologies) at 50°C for 2 mins and then 50 cycles of 95°C for 15 secs and 60°C for 45 secs, using the following primers: ORF28 forward, CGAACACGTTCCCCATCAA; ORF28 reverse, CCCGGCTTTGTTAGTTTTGG; Albumin forward, TTTGCAGATGTCAGTGAAAGAGA; Albumin reverse, TGGGGAGGCTATAGAAAATAAGG [86]. Relative viral genome copies were determined by normalising ORF28 levels to Albumin, and values were depicted as fold change over the initial time point (4 hpi).

### Statistical analyses

Statistics were calculated with GraphPad Prism (version 7; GraphPad Software). Tests for normality were performed, as well as the number of comparisons considered in order to determine the appropriate statistical test to use in each case.

### Ethics statement

All blood work was performed in accordance with The University of Sydney ethics approval. All donors provided written informed consent.

## Supporting information

S1 Fig VZV-GFP infects human peripheral blood NK cells, CD3^+^CD56^+^ lymphocytes, and T cells. Healthy human donor PBMCs were inoculated with ARPE-19 epithelial cells mock infected or infected with VZV expressing GFP (VZV-GFP) for 2 days then analysed for infection by flow cytometry. (A) Representative flow cytometry plots of live T cells (CD3^+^CD56^–^), CD3^+^CD56^+^ lymphocytes, and NK cells (CD3^–^CD56^+^), examining GFP expression. (B) Frequencies of live GFP^+^ T cells, CD3^+^CD56^+^ lymphocytes, and NK cells (n = 3). Colours represent individual donors, and bars indicate mean. *p < 0.05 (Friedman test with Dunn’s multiple comparisons test).
(TIF)

S2 Fig Cell-free VARIVAX VZV infects human peripheral blood NK cells. Healthy human donor PBMCs were inoculated with VARIVAX vaccine (MOI 0.001) for 2 days then analysed for infection by flow cytometry. Shown are example flow cytometry plots of live NK cells (CD3^–^CD56^+^) examining surface VZV gE:gI expression (A) or Fluorescence Minus One (FMO) control for gE:gI staining (B) (n = 3).
(TIF)

S3 Fig Increase in viral genome copies in VZV cultured NK cells over time. Healthy human donor PBMCs were infected with VZV for 4 hours and then NK cells (CD3^–^CD56^+^) were isolated by FACS sorting. A sample of isolated NK cells were harvested immediately following sorting, while remaining NK cells were further cultured at 37°C and harvested at the specified time points post infection. DNA was subsequently extracted and qPCR performed, quantifying VZV ORF28 and albumin. Viral genome copies were calculated as ORF28/albumin and depicted as fold change over the initial time point (4 hpi). Data from two donors (A & B) are shown.
(TIF)

S4 Fig Transmission of infection from VZV cultured NK cells stripped with citrate buffer. (A) NK cells (CD3^–^CD56^+^) were FACS sorted from healthy human donor PBMCs following VZV infection for 1 day. Isolated NK cells were subsequently washed with citrate buffer and PBS before being added to ARPE-19 epithelial cell monolayers. After 4 days in culture, monolayers were observed under light microscope for CPE. Plaques are indicated by arrowheads. One representative experiment of two is shown.
(TIF)
