# Supplementary figures and images for: Varicella zoster virus productively infects human natural killer cells and manipulates phenotype
Source: PLoS Pathog. 2018 Apr 30;14(4):e1006999. doi: 10.1371/journal.ppat.1006999 (PMC5953475; doi:10.1371/journal.ppat.1006999)

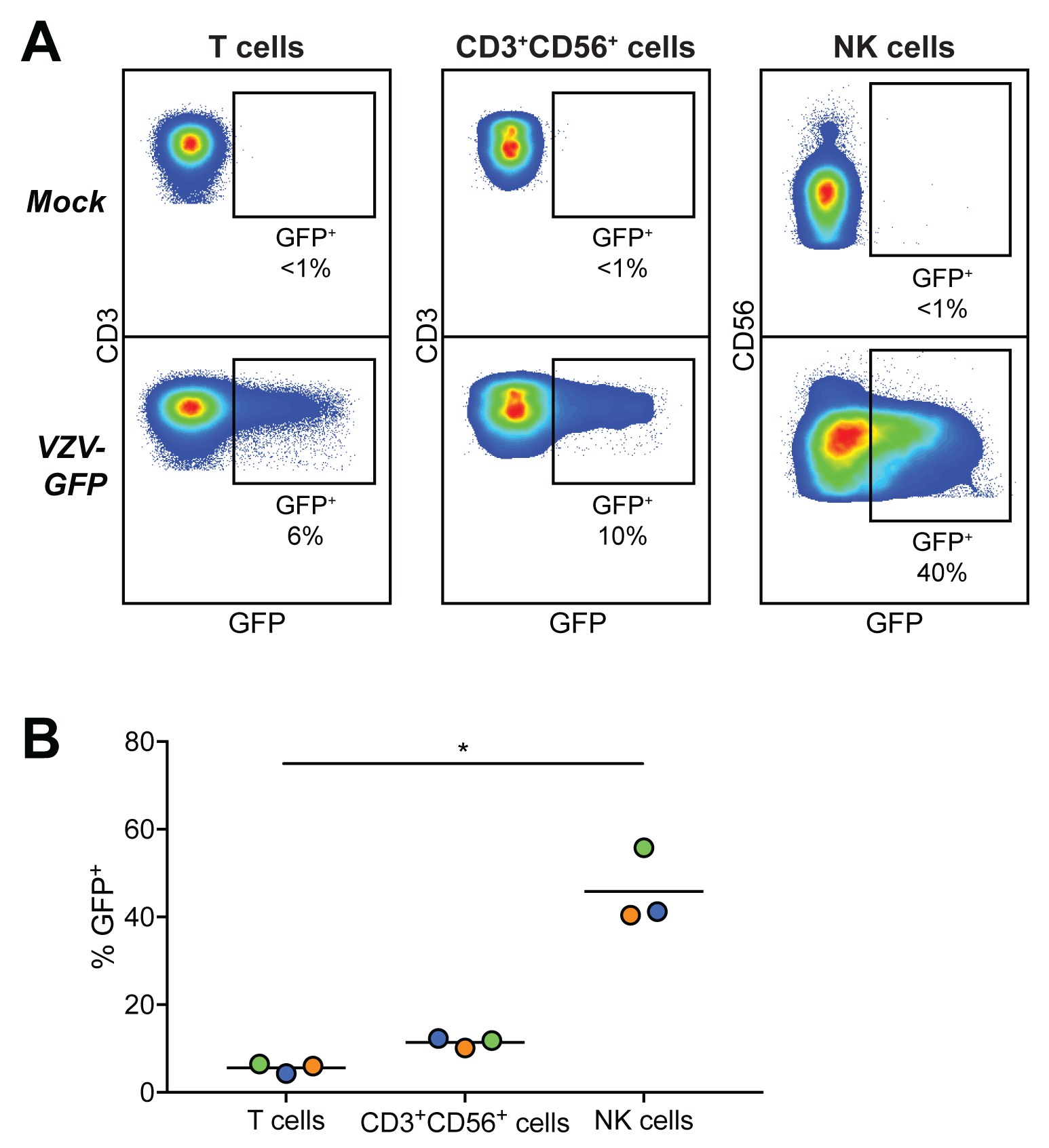

Supplement: S1 Fig — Healthy human donor PBMCs were inoculated with ARPE-19 epithelial cells mock infected or infected with VZV expressing GFP (VZV-GFP) for 2 days then analysed for infection by flow cytometry. (A) Representative flow cytometry plots of live T cells (CD3+CD56–), CD3+CD56+ lymphocytes, and NK cells (CD3–CD56+), examining GFP expression. (B) Frequencies of live GFP+ T cells, CD3+CD56+ lymphocytes, and NK cells (n = 3). Colours represent individual donors, and bars indicate mean. *p < 0.05 (Friedman test with Dunn’s multiple comparisons test). (TIF) [file ppat.1006999.s001.tif]

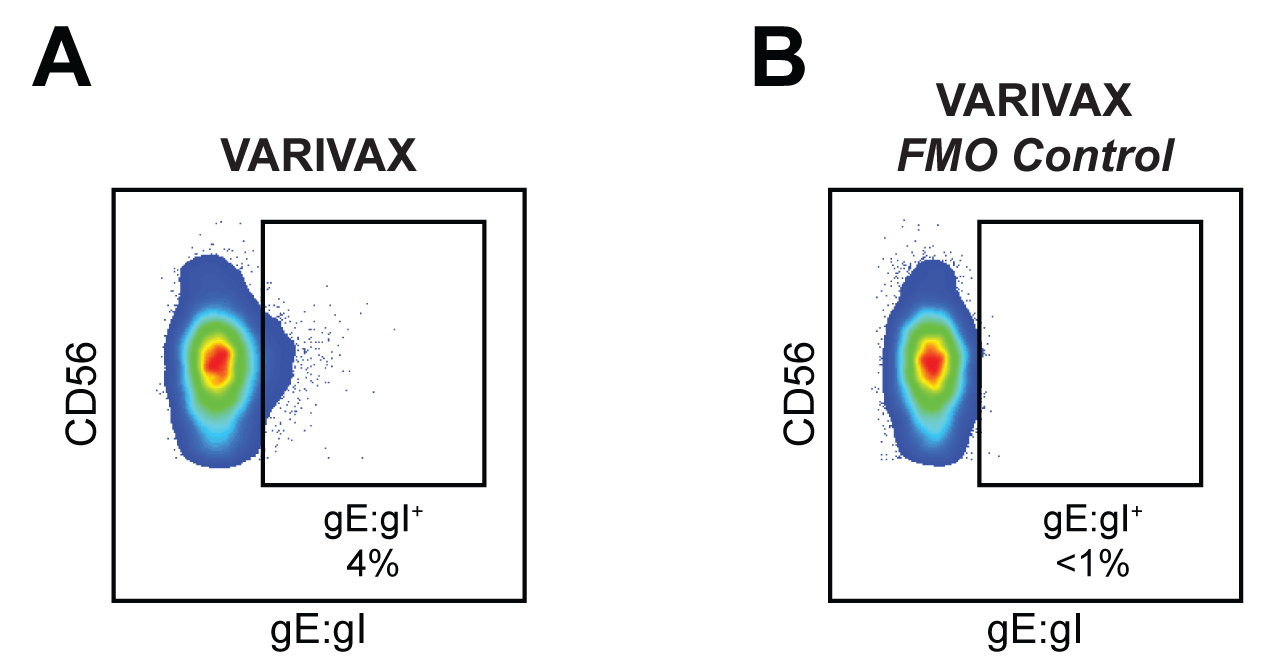

Supplement: S2 Fig — Healthy human donor PBMCs were inoculated with VARIVAX vaccine (MOI 0.001) for 2 days then analysed for infection by flow cytometry. Shown are example flow cytometry plots of live NK cells (CD3–CD56+) examining surface VZV gE:gI expression (A) or Fluorescence Minus One (FMO) control for gE:gI staining (B) (n = 3). (TIF) [file ppat.1006999.s002.tif]

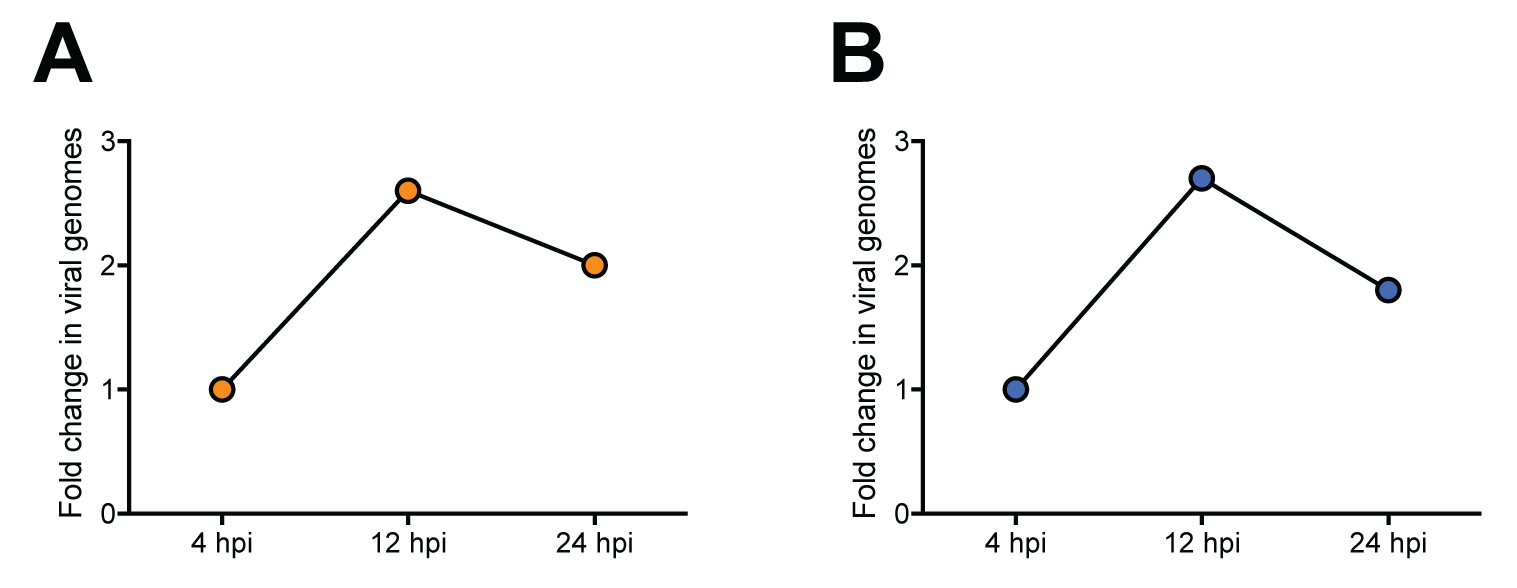

Supplement: S3 Fig — Healthy human donor PBMCs were infected with VZV for 4 hours and then NK cells (CD3–CD56+) were isolated by FACS sorting. A sample of isolated NK cells were harvested immediately following sorting, while remaining NK cells were further cultured at 37°C and harvested at the specified time points post infection. DNA was subsequently extracted and qPCR performed, quantifying VZV ORF28 and albumin. Viral genome copies were calculated as ORF28/albumin and depicted as fold change over the initial time point (4 hpi). Data from two donors (A & B) are shown. (TIF) [file ppat.1006999.s003.tif]

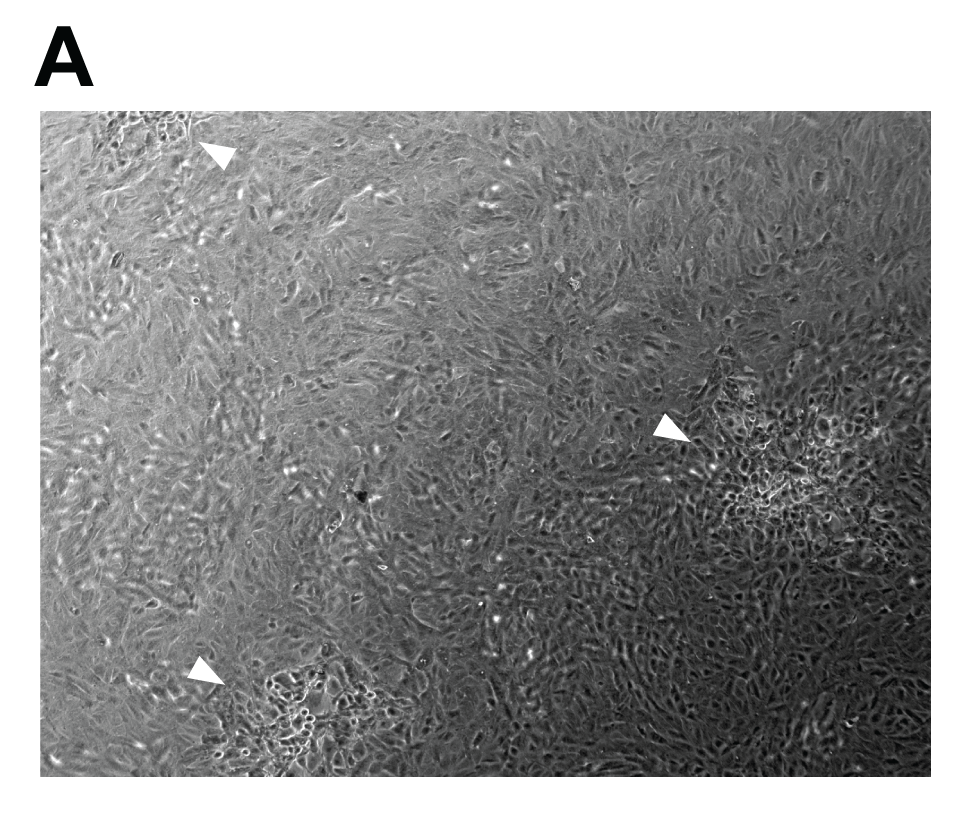

Supplement: S4 Fig — (A) NK cells (CD3–CD56+) were FACS sorted from healthy human donor PBMCs following VZV infection for 1 day. Isolated NK cells were subsequently washed with citrate buffer and PBS before being added to ARPE-19 epithelial cell monolayers. After 4 days in culture, monolayers were observed under light microscope for CPE. Plaques are indicated by arrowheads. One representative experiment of two is shown. (TIF) [file ppat.1006999.s004.tif]
